# Redefining High Emergency Department Utilization for Sickle Cell Disease

**DOI:** 10.1001/jamanetworkopen.2025.13361

**Published:** 2025-06-02

**Authors:** Paula Tanabe, Wei Pan, Audrey L. Blewer, Daniel Hatch, Camila Reyes, Lauren Siewny, John J. Strouse, Matthew Young, Mariam Kayle

**Affiliations:** 1 Duke University School of Nursing, Durham, North Carolina; 2 Duke University School of Medicine, Durham, North Carolina; 3 Department of Family Medicine and Community Health, Duke University School of Medicine, Durham, North Carolina; 4 Department of Population Health Sciences, Duke University School of Medicine, Durham, North Carolina; 5 Duke Office of Clinical Research, Duke University School of Medicine, Durham, North Carolina; 6 Department of Emergency Medicine, Duke University School of Medicine, Durham, North Carolina; 7 Division of Hematology, Duke University School of Medicine, Durham, North Carolina; 8 Division of Pediatric Hematology, Duke University School of Medicine, Durham, North Carolina; 9 Wake Emergency Physicians, Professional Association, Cary, North Carolina

## Abstract

**Importance:**

Sickle cell disease (SCD) is a complex hemoglobinopathy. Vaso-occlusive episodes are the primary cause of emergency department (ED) utilization among individuals with SCD. Literature lacks a standardized definition for high ED utilization.

**Objective:**

To explore ED utilization, redefine high ED utilization, and describe factors associated with super-high ED utilization among individuals with SCD.

**Design, Setting, and Participants:**

This retrospective cohort study analyzed the North Carolina Hospital Discharge Datasets (2013-2019). Participants were included if they had SCD, defined as at least 3 SCD visits (ED, inpatient, or outpatient surgery) in a rolling 5-year period. All age groups, sexes, and payers, regardless of state of residence, were included. Data were analyzed from July 2023 to August 2024.

**Exposures:**

Variables included sex, race, ethnicity, age, age at death, distance (in miles) to the closest SCD center, number of annual ED visits, and social vulnerability index (SVI).

**Main Outcomes and Measures:**

To determine ED utilization categories, first the distribution of people based on the number of annual ED visits was examined, then the categories across years were identified to determine the data-informed cutoff for each category. Univariate analysis determined differences between participants based on the ED utilization category using χ^2^ tests of independence or analysis of variance, as applicable. Descriptive statistics were conducted to describe characteristics of utilization in the sample and by ED utilization group. A parsimonious multinomial regression was conducted using significant factors from the univariate analysis.

**Results:**

The cohort included 9964 unique patients (5364 [53.83%] female; mean [SD] age, 24.49 [17.54] years), including 9355 Black patients (93.89%), with 100 188 total ED visits from 2013 to 2019. ED visits were categorized into 4 levels: low (0-1 visits per year), moderate (2-9 visits per year), high (10-32 visits per year), and super high (≥33 visits per year). A small subset (178 patients [1.79%]) exhibited super-high ED utilization, contributing disproportionately to the total number of ED visits. Older age, younger age for in-facility deaths, and higher SVI were significantly associated with higher ED utilization. Patients with high utilization were more likely to die, die younger (eg, median [IQR] age at death, 33.0 [30.0-44.0] years in the super-high utilization group vs 50 [38.0-61.0] years in the moderate utilization group), use multiple EDs (eg, 93.8% of participants in the super-high utilization group vs 40.08% of participants in the moderate utilization group), and reside in counties more disadvantaged on socioeconomic and transportation characteristics.

**Conclusions and Relevance:**

This cohort study of 7 years of North Carolina Hospital Discharge Data described 4 new categories of ED utilization in SCD. These categories could be used to reframe how high ED use is determined.

## Key Points

**Question:**

How should high emergency department utilization for individuals with sickle cell disease (SCD) be defined?

**Findings:**

This cohort study including 9964 unique patients with SCD analyzed 7 years of all ED visits in North Carolina, using longitudinal data to define categories of ED visits. Four categories of ED use for individuals with SCD were identified: low, 0 to 1 visit in every year (34% of people); moderate, 2 to 9 visits in any year (56.5% of people); high, 10 to 32 visits in any year (7.6% of people); and super high, 33 or more visits in any year (1.79% of people); individuals in the moderate, high, and super high categories were older, more likely to die and die younger, and have visited multiple EDs, and they had a higher Social Vulnerability Index.

**Meaning:**

These findings support using new data-based categories to rethink how high ED use is defined.

## Teaser

This cohort study describes characteristics of emergency department utilization and redefines emergency department utilization categories in a longitudinal cohort of patients with sickle cell disease in North Carolina.

## Introduction

Sickle cell disease (SCD) is a complex genetic hemoglobinopathy affecting approximately 100 000 persons in the US.^1^ It is associated with a severely shortened lifespan. A 2020 report estimated the median age of death for individuals with SCD is 43 years.^2^ The most frequent complication experienced by individuals with SCD is severe painful events, referred to as vaso-occlusive episodes (VOEs). An increased number of VOEs per year has been found to be associated with an increased risk of mortality, which highlights the need for rapid evaluation and treatment.^3^ Between 1999 and 2020, there were an estimated 4.9 million ED visits in the US for SCD, with 75% for treatment of VOE.^4^ However, there is large variability in the number of ED visits per patient. Furthermore, high ED utilization could reinforce an ED clinician’s perception of opioid addiction and impact care, despite a lack of data to support this.^5^ Importantly, individuals with SCD should seek emergency care when needed. However, for patients with very high ED use, it is important to understand the reasons for high utilization. Often social determinants of health, disease severity, behavioral health comorbidities, and lack of outpatient care may influence ED use.

There is no clear consensus of how many ED visits per patient per year meet the definition of high ED utilization. Definitions range from 2 or more in a Saudi Arabian cohort^6^ to 3 to 24 or more visits per year in US-based reports, with the most commonly cited as 3 to 6 ED visits per year.^3,7,8,9,10,11,12,13,14,15,16,17,18,19,20,21,22,23^ A few reports have demonstrated a wide range in the number of ED visits per individual patient. In a 2005 California cohort, 53% of individuals had no ED visits and 35% of individuals had 1 to 3 visits.^20^ Importantly, in this same cohort, 122 individuals had 11 or more ED visits and up to 185 visits in a single year.^20^ Others have reported a range of up to 311 ED visits per individual in 1 year.^21^ As far back as 1989, Yang and colleagues^24^ identified 4 patients with 31 to 100 ED visits in 1 year.^24^ The definitions of high ED use have been determined arbitrarily.

Given the extraordinarily wide range in definitions of high ED utilization in the literature, our clinical experiences of a much higher number of ED visits from a very small number of patients, and the negative effect this may have on biases toward persons with SCD, we felt it was important to redefine the number of individuals with high ED utilization in North Carolina over a 7-year period. Understanding actual utilization based on data for individuals with SCD is important as it will allow for early identification of potential changes in utilization for an individual patient. Clinicians can then begin to investigate reasons for utilization changes with the patient earlier. We used the all-payer North Carolina Hospital Discharge Datasets from 2013 to 2019 to explore ED utilization and to redefine high ED utilization for individuals with SCD. We also described factors associated with high ED utilization in the cohort across time. The overall goal of this analysis was to better understand how many visits would meet the definition of high ED utilization and who were the individuals with high ED utilization.

## Methods

### Study Design and Setting

This was a retrospective cohort study using the statewide, all-payer North Carolina Hospital Discharge Data between October 1, 2012, and December 31, 2020. We used the Strengthening the Reporting of Observational Studies in Epidemiology (STROBE) reporting guideline for cohort studies. The institutional review board at Duke University approved the study with a waiver of informed consent because the research was determined to involve no more than minimal risk, could not be performed without the waiver, and did not affect the rights or welfare of individuals.

### Participants and Data Source

Participants were included if they had SCD, defined as 3 or more visits (ED, inpatient, or outpatient surgery) with an *International Classification of Diseases, Ninth Revision, Clinical Modification (ICD-9-CM)* or *International Statistical Classification of Diseases, Tenth Revision, Clinical Modification (ICD-10-CM)* for SCD in a rolling 5-year period between October 1, 2012, and December 31, 2020 (eFigure in Supplement 1). This definition has demonstrated 96% sensitivity and 76.5% specificity in identifying SCD in an administrative database.^25^ Participants were included regardless of the state of residence. The North Carolina Hospital Discharge Data are limited datasets. Patients were identified across the dataset by a unique identifier combining date of birth, sex, and 5-digit zip code and were followed-up across calendar years for the duration of the study.

### Outcome Variables

The main outcome was the annual number of ED visits between January 1, 2013, and December 31, 2019. ED visits were defined as any ED visit, which included both treat-and-release ED discharges and admission to the hospital.

### Exposure Variables

Demographic variables, including sex, race, ethnicity, and age, were extracted from visit-level data. Race and ethnicity were reported as recorded in the database. Race was categorized as Black or African American, declined or unavailable, other race (eg, American Indian, Asian, Native Hawaiian or Pacific Islander, White, other), or unknown. Ethnicity was categorized as Hispanic, non-Hispanic, or unknown. Age was calculated as age at the first visit in the full dataset. In-facility death was determined based on expired disposition code at the visit level and did not capture deaths outside of a health care facility. Age at death was calculated as age at discharge with an expired disposition code. Death and age at death are important to examine because they are measures of disease severity. Understanding the association can heighten clinician concern when individuals begin to have an increasing number of ED visits. Distance in miles between the participant’s 5-digit zip code and the 5-digit zip code of the closest SCD center was calculated. Multi-ED use (visits at multiple ED facilities) was operationalized by calculating the number of ED facilities a participant visited per year.

The 2020 state-ranked Social Vulnerability Index (SVI) was used to measure county-level socioeconomic factors associated with the number of ED visits.^26^ The 2020 SVI ranks counties within a state on 16 social factors under 4 themes: socioeconomic status, household characteristics, racial and ethnic minority status, and housing type and transportation. Percentile ranking values range from 0 to 1, with higher values indicating more vulnerability. An overall SVI and theme-level scores were calculated for participants who had a North Carolina address.

### Statistical Analysis

Descriptive statistics were used to describe sample characteristics and ED visits. To develop the categories of ED visit utilization, we first examined the distribution of people based on the number of visits for each year between 2013 and 2019. We then identified utilization categories across years to determine the data-informed cutoff for each category. Low utilization was defined as the 50th percentile or lower; moderate utilization, between the 51st and 94th percentiles; high utilization, between the 95th and 98th percentiles; and super high utilization, the 99th percentile or higher. These cut points were selected to identify the majority of patients (51st percentile) and the extremes. Similar to Carroll et al,^27^ who categorized individuals based on hospitalizations, we classified participants into mutually exclusive ED utilization categories, giving the priority to higher ED utilization categories. While Carroll et al^27^ identified individuals with continuous and moderate hospital utilization over the 5-year period, individuals were ultimately categorized when they experienced 4 or more hospitalizations in any 1 of the 5-year periods. We believe it is equally important to identify transient vs only chronic high utilization, as this allows early identification of individuals who may need more intervention, and it is not possible to predict when utilization may decline. Finally, we defined low utilization as 0 to 1 visit in every year vs the other categories in any year because the focus of this study was on high utilization. We a priori discussed this and felt it was important to identify consistent low utilization but recognize high utilization fluctuates over time. Analysis was conducted to determine whether there were differences between participants based on the ED utilization category, using χ^2^ tests of independence for categorical characteristics and analysis of variance for continuous characteristics. Descriptive statistics were also conducted to describe characteristics of utilization, in the sample overall and by ED utilization group. These characteristics included total number of ED visits across years and patients, mean and median ED visits per person per year, number of all hospitalizations, number of ED visits that resulted in hospitalizations, ED to hospitalization length of stay, and primary payer of ED visits. We also collapsed age into fewer categories for analytical modeling due to power and sample size limitations of the more granular age bins in descriptive statistics.

Multinomial logistic regression was conducted for a full model with factors identified in the univariate analysis and a parsimonious model with a stepwise selection with only significant factors, both without variables that were multicollinearly related with other factors (eg, race and ethnicity related with SVI) or highly associated with the outcome (eg, mean number of years in study and visited multiple EDs in any year). This analysis was conducted to examine the association between multiple factors and ED utilization categories. Finally, a subanalysis was performed to determine the number of individuals and related ED visits per year for individuals who did not reside in North Carolina.

All analyses were conducted using SAS software version 9.4 (SAS Institute). Statistical significance was 2-tailed and set at α = .05. Data were analyzed from July 2023 to August 2024.

## Results

### Sample Demographics

During the study period, a total of 9964 unique individuals with SCD (5364 [53.83%] female; mean [SD] age, 24.49 [17.54] years) met the inclusion criteria, including 9355 Black patients (93.89%) (Table 1). A total of 269 patients (2.7%) died in a health care facility during the study period, and median (IQR) age at death among these individuals was 46 (31-58) years. In general, participants lived close to SCD centers (mean [SD], 33.87 [70.41] miles), and among participants who lived in North Carolina, 6728 (67.52%) lived in counties with high or very high social vulnerability (overall SVI, >0.5). Specifically, almost 50% of participants lived in counties that were disadvantaged on the housing type and transportation theme and 60% lived in counties that were disadvantaged on the household characteristics theme (Table 1).

**Table 1.** Characteristics of People With SCD by ED Utilization, 2013-2019

Characteristic	Individuals, No. (%)					*P* value^a^
	Overall (N = 9964)	Individuals by utilization				
		Low (0-1 ED visits in each year) (n = 3397 [34.09%])	Moderate (2-9 visits in any year (n = 5631 [56.51%])	High (10-32 visits in any year) (n = 758 [7.61%])	Super high (≥33 visits in any year) (n = 178 [1.79%])	
Sex						
Male	4600 (46.17)	1553 (45.72)	2591 (46.01)	370 (48.81)	86 (48.31)	.42
Female	5364 (53.83)	1844 (54.28)	3040 (53.99)	388 (51.19)	92 (51.69)	
Race						
Black or African American	9355 (93.89)	3098 (91.20)	5349 (94.99)	731 (96.44)	177 (99.44)	<.001
Declined or unavailable	223 (2.24)	125 (3.68)	92 (1.63)	<11^b^	<11^b^	
Other^c^	386 (3.87)	174 (5.12)	190 (3.37)	>16^b^	<11^b^	
Ethnicity						
Hispanic	189 (1.90)	73 (2.15)	107 (1.90)	<11^b^	<11^b^	<.001
Non-Hispanic	9380 (94.14)	3118 (91.79)	5354 (95.08)	732 (96.57)	176 (98.88)	
Unknown	395 (3.96)	206 (6.06)	170 (3.02)	>15^d^	<11^d^	
Age, y						
Mean (SD), y	24.49 (17.54)	23.02 (19.28)	24.95 (17.30)	27.09 (11.08)	26.74 (7.46)	<.001
<10	2395 (24.04)	1076 (31.68)	1280 (22.73)	39 (5.15)	0 (0)	<.001
10-19	1775 (17.81)	697 (20.52)	918 (16.30)	135 (17.81)	25 (14.04)	
20-29	2262 (22.70)	479 (14.10)	1386 (24.61)	294 (38.79)	103 (57.87)	
30-39	1556 (15.62)	422 (12.42)	918 (16.30)	180 (23.75)	36 (20.22)	
40-49	1021 (10.25)	315 (9.27)	604 (10.73)	90 (11.87)	12 (6.74)	
50-59	584 (5.86)	231 (6.80)	334 (5.93)	>11^b^	<11^b^	
≥60	371 (3.72)	177 (5.21)	191 (3.39)	<11^b^	<11^b^	
Time in the study, mean (SD), y	2.64 (1.73)	1.55 (0.81)	2.77 (1.67)	3.98 (2.00)	5.02 (1.89)	<.001
Infacility death	269 (2.70)	41 (1.21)	175 (3.11)	>42^b^	<11^b^	<.001
Age at death, median (IQR), y^d^	46.0 (31.0-58.0)	48.0 (23.0-62.0)	50.0 (38.0-61.0)	32.0 (28.0-44.0)	33.0 (30.0-44.0)	<.001
Distance to closest SCD center from residence zip code, mean (SD), miles	33.87 (70.41)	33.36 (88.16)	34.78 (61.97)	31.09 (40.53)	26.90 (25.46)	.26
Visited multiple EDs in any year	3033 (30.44)	NA^e^	2257 (40.08)	609 (80.34)	167 (93.82)	<.001^e^
EDs visited, mean (SD), No.	1.83 (0.76)	NA^e^	1.65 (0.44)	2.13 (0.88)	3.06 (1.67)	<.001
SVI^f^						
Overall, mean (SD)	0.56 (0.24)	0.55 (0.23)	0.57 (0.24)	0.57 (0.23)	0.56 (0.22)	<.001
Overall score >0.5	6728 (67.52)	2191 (64.50)	3894 (69.15)	522 (68.87)	121 (67.98)	<.001
Socioeconomic status >0.5	3302 (33.14)	1022 (30.09)	1970 (34.98)	252 (33.25)	58 (32.58)	<.001
Household characteristics >0.5	5991 (60.13)	1915 (56.37)	3504 (62.23)	465 (61.35)	107 (60.11)	<.001
Racial and ethnic minority status >0.5	8176 (82.06)	2781 (81.87)	4622 (82.08)	621 (81.93)	152 (85.39)	.69
Housing type and transportation >0.5	4926 (49.44)	1567 (46.13)	2875 (51.06)	390 (51.45)	94 (52.81)	<.001

Abbreviations: ED, emergency department; NA, not applicable; SCD, sickle cell disease; SVI, Social Vulnerability Index.

^a^ Assessed using χ^2^ or analysis of variance, as appropriate.

^b^ Cell counts with <11 are suppressed.

^c^ Other race include American Indian, Asian, Native Hawaiian or Pacific Islander, White, and other, as reported in the database.

^d^ Age at death calculated from discharge date where visit disposition is expired minus the date of birth.

^e^ Given that the low utilization group could not have had visits at multiple EDs, this group was excluded from the univariate analysis.

^f^ Score range from 0 to 1, with higher score indicating higher vulnerability. The threshold of greater than 0.5 reflects high and very high social vulnerability. SVI was calculated only for people with a North Carolina zip code (8201 individuals [95.5%]).

Table 2 provides the ED visits overall and by utilization groups during the study period. Between 2013 and 2019, 9964 participants contributed 100 188 ED visits, with a median of 1.5 (0.9-3.0) visits per year. Using our predefined cutoffs, low utilization was 0 to 1 ED visits in every year (3397 participants [34.09%]); moderate utilization, 2 to 9 visits in any year (5631 participants [56.51%]); high utilization, 10 to 32 visits in any year (758 participants [7.61%]); and super-high utilization, 33 or more visits in any year (178 participants [1.79%]). Importantly, less than 10% of participants in the study contributed 55% of all ED visits. Specifically, 178 participants (1.79%) contributed 27 358 ED visits (27.3%) and 758 individuals (7.6%) contributed an additional 28 124 ED visits (28.1%). Almost one-third of all ED visits (31 406 visits [31.35%]) resulted in hospitalizations, with a mean (SD) length of stay of 5.1 (4.6) days. Most ED visits were paid by Medicaid (47 689 visits [47.60%]) and Medicare (32 099 visits [32.04%]). Table 3 reports the ED visits per year for each utilization cohort. The number of unique patients per year and number of visits was consistent across the study years.

**Table 2.** ED Visits by Utilization Group, 2013-2019

Measure	ED visits, No. (%)				
	Overall (N = 100 188)	Utilization			
		Low (0-1 ED visits in each year) (n = 3124 [3.12%])	Moderate (2-9 visits in any year (n = 41 582 [41.50%])	High (10-32 visits in any year) (n = 28 124 [28.07%])	Super high (≥33 visits in any year) (n = 27 358 [27.31%])
Visits per person per year, median (IQR), No.	1.5 (0.9-3.0)	0.5 (0.3-1.0)	2.0 (1.2-3.0)	8.3 (5.8-11.5)	26.2 (19.0-35.7)
Inpatient hospitalizations, No.^a^	40 493	2290	19 260	12 199	6744
ED visits that resulted in hospitalization	31 406 (31.35)	968 (30.99)	14 224 (34.21)	10 235 (36.39)	5979 (21.85)
ED to hospitalization LOS, mean (SD), d	5.08 (4.64)	4.70 (7.00)	4.91 (4.61)	5.41 (4.74)	4.97 (4.00)
Primary payer					
Self-pay	5511 (5.50)	252 (8.07)	3378 (8.12)	1219 (4.33)	662 (2.42)
Private	13 136 (13.11)	897 (28.71)	7696 (18.51)	2676 (9.52)	1867 (6.82)
Medicare	32 099 (32.04)	454 (14.53)	9245 (22.23)	11 317 (40.24)	11 083 (40.51)
Medicaid	47 689 (47.60)	1397 (44.72)	20 070 (48.27)	12 606 (44.82)	13 616 (49.77)
Other^b^	1452 (1.45)	106 (3.39)	1024 (2.46)	223 (0.79)	99 (0.36)
Unknown	301 (0.30)	18 (0.58)	169 (0.41)	83 (0.30)	31 (0.11)

Abbreviations: ED, emergency department; LOS, length of stay.

^a^ Includes direct admission or admission from the ED.

^b^ Other insurance is not Medicaid, Medicare, private insurance, or self-pay and includes nonfederal or federal programs, such as Department of Veterans Affairs, Tricare, Title V, and other programs, such as workers’ compensation.

**Table 3.** Characteristics of SCD Cohort and ED Visits by Year, 2013-2019

Measure	By year							Cumulative, 2013-2019
	2013	2014	2015	2016	2017	2018	2019	
Unique patients with SCD, No.	4069	4480	4726	4654	4706	4784	4953	9964^a^
Unique patients with ≥1 ED visit in each year, No. (%)	2841 (69.82)	3158 (70.49)	3289 (69.59)	3299 (70.89)	3340 (70.97)	3358 (70.19)	3368 (68.00)	8588 (86.20)^a^
ED visits, No.	12 800	14 066	14 486	14 775	14 606	14 475	14 980	100 188
ED visits per person, No.								
Mean (SD) [range]	4.51 (8.84) [1-189]	4.45 (9.97) [1-218]	4.42 (8.64) [1-167]	4.48 (8.40) [1-196]	4.37 (8.05) [1-122]	4.31 (7.41) [1-114]	4.45 (8.49) [1-136]	3.6 (5.5) [1-131]^b^
Median (IQR)	2 (1-4)	2 (1-4)	2 (1-4)	2 (1-4)	2 (1-4)	2 (1-4)	2 (1-4)	2 (1.3-3.5)^b^

Abbreviations: ED, emergency department; SCD, sickle cell disease.

^a^ The numbers reported in these cells are not sum totals and provide the unique numbers of people in the study between 2013 and 2019.

^b^ Calculated per person per ED visit years.

The proportion of Black and non-Hispanic participants differed by ED utilization group in that participants in the super-high utilization group had a higher proportion of participants in these racial and ethnic categories compared with the other utilization groups (Table 1). Participants in the high and super-high ED utilization groups were older and had more years in the study compared with the low and moderate ED utilization groups. Although almost 80% of the 269 in-facility deaths occurred among the low (41 patients) and moderate (175 patients) ED utilization groups, participates in the high and very high ED utilization groups died younger (median [IQR] age at death, 32.0 [28.0-44.0] years and 33.0 [30.0-44.0] years, respectively) compared with low and moderate ED utilization groups (median [IQR] age at death, 48 [23.0-62.0] years and 50 [38.0-61.0] years, respectively). A higher proportion of participants in the high (609 participants [80.34%]) and super-high (167 participants [93.8%]) utilization groups had visits in multiple EDs compared with the moderate utilization group (2257 participants [40.08%]). Compared with participants in the low utilization group, a higher proportion of participants in the moderate, high, and super-high utilization groups lived in disadvantaged areas on socioeconomic status, household characteristics and housing type and transportation themes (Table 1). There were no differences among the utilization groups based on sex or how close they lived to the nearest SCD center.

Table 4 reports estimated odds ratios (ORs) from our multinomial logistic regression analysis. Both the full and parsimonious models found that age group, died in a facility, and SVI were significantly associated with ED visit grouping. Taking the parsimonious model as an example, compared with the youngest age group (age <19 years), the older age groups were significantly more likely to have moderate (age 19-39 years: OR, 2.07; 95% CI, 1.88-2.29; age ≥40 years: OR, 1.22; 95% CI, 1.09-1.37) or high (age 19-39 years: OR, 6.09; 95% CI, 4.98-7.46; age ≥40 years: OR, 1.57; 95% CI, 1.20-2.05) ED utilization vs low ED utilization. Notably, the young adult group (age 19-39 years) was more than 16 times likely to have super high ED utilization (OR, 16.44; 95% CI, 9.75-27.72) vs low ED utilization. If a participant died in a facility, they were significantly more likely to have super high, high, or moderate ED utilization vs low ED utilization (eg, high utilization: OR, 5.18; 95% CI, 3.32-8.08). As for SVI, patients living in the areas with SVI more than the median score (>50%) in household characteristics or housing type and transportation were significantly more likely to have moderate (household characteristics: OR, 1.25; 95% CI, 1.15-1.37; housing type and transportation: OR, 1.16; 95% CI, 1.06-1.26) or high (household characteristics: OR, 1.24; 95% CI, 1.04-1.47; housing type and transportation: OR, 1.19; 95% CI, 1.01-1.41) ED utilization vs low ED utilization.

**Table 4.** ORs From the Parsimonious Multinomial Logistic Regression on ED Visits, 2013-2019

Variable	OR (95% CI) of ED utilization group^a^		
	Moderate: 2-9 ED visits in any year (n = 5631)	High: 10-ED 32 visits in any year (n = 758)	Super high: ≥33 ED visits in any year (n = 178)
Age range, y			
<19	1 [Reference]	1 [Reference]	1 [Reference]
19-39	2.07 (1.88-2.29)	6.09 (4.98-7.46)	16.44 (9.75-27.72)
≥40	1.22 (1.09-1.37)	1.57 (1.20-2.05)	1.90 (0.92-3.93)
In-facility death			
No	1 [Reference]	1 [Reference]	1 [Reference]
Yes	2.50 (1.76-3.53)	5.18 (3.32-8.08)	3.50 (1.52-8.06)
SVI, household characteristics >0.5^b^			
No	1 [Reference]	1 [Reference]	1 [Reference]
Yes	1.25 (1.15-1.37)	1.24 (1.04-1.47)	1.18 (0.85-1.63)
SVI, housing type and transportation >0.5^b^			
No	1 [Reference]	1 [Reference]	1 [Reference]
Yes	1.16 (1.06-1.26)	1.19 (1.01-1.41)	1.31 (0.95-1.80)

Abbreviations: ED, emergency department; OR, odds ratio; SVI, Social Vulnerability Index.

^a^ For all analyses, the comparison group was individuals with low utilization (0-1 ED visits in each year).

^b^ Score range from 0 to 1, with higher score indicating higher vulnerability. The threshold of greater than 0.5 reflects high and very high social vulnerability.

To understand where all patients lived, we explored all ED visits that occurred in North Carolina, regardless of state of residence. We identified 387 out-of-state participants who resided in 24 states and the District of Columbia, with 3113 ED visits in North Carolina (Table 5). Of these out-of-state participants, 43 had at least 10 ED visits in North Carolina in any year. While most patients were from surrounding states, such as South Carolina and Virginia, many were not. Similar to participants in North Carolina, approximately one-third of these visits resulted in hospitalizations; the mean number of ED visits per year was also similar (Table 5). Most of the ED visits were paid for by Medicaid (1320 visits [42.40%]) and Medicare (742 visits [23.84%]).

**Table 5.** Characteristics of Out-of-State Participants With ED Visits, 2013-2019

Out of state cohort demographic characteristics	No. (%)
**Patient-level data (n= 387)**	
With <10 ED visits in all years	344 (88.89)
ED visits per person per ED visit years (among participants with <10 visits), No.	
Mean (SD) [range]	1.88 (1.58) [0.14-9.00]
Median (IQR)	1.41 (0.67-3.00)
With ≥10 ED visits in any year	43 (11.11)
ED visits per person per ED visit years (among participants with ≥10 visits), No.	
Mean (SD) [range]	11.04 (7.12) [3.00-39.86]
Median (IQR)	10.00 (5.67-14.00)
Sex	
Male	186 (48.06)
Female	201 (51.94)
Unknown	0
Race	
Black or African American	380 (98.19)
Other race^a^	<11
Unknown	<11
Ethnicity	
Non-Hispanic	373 (96.38)
Hispanic or unknown	14 (3.73)
Age, y	
Mean (SD)	26.45 (14.99)
Range	
<10	53 (13.70)
10-19	65 (16.80)
20-29	123 (31.78)
30-39	70 (18.09)
40-49	43 (11.11)
≥50	33 (8.53)
Time in study, mean (SD), y	2.06 (1.41)
In-facility death, No. (%)	
No	376 (97.16)
Yes	11 (2.84)
Age at death, mean (SD), y	41.55 (13.37)
State of residence, No. (%)^b^	
SC	202 (52.20)
VA	57 (14.73)
CT, MA, PA, NJ	24 (6.20)
MD, DC, AL, LA, MS, TN, TX, KY	24 (6.20)
IL, MI, WI, OH, KS, NM, CA, AK	24 (6.20)
NY	23 (5.94)
GA	20 (5.17)
FL	13 (3.36)
**Visit-level data (n = 3113)**	
ED visits per person per year, mean (SD), No.	2.90 (4.00)
All inpatient visits^c^	1421
ED visits that resulted in hospitalization	1002 (32.19)
ED to inpatient LOS, mean (SD), d	5.17 (4.49)
Primary payer of ED visits	
Self-pay	258 (8.29)
Private	759 (24.38)
Medicare	742 (23.84)
Medicaid	1320 (42.40)
Other	14 (0.45)
Unknown	20 (0.64)

Abbreviations: AK, Alaska; AL, Alabama; CA, California; CT, Connecticut; DC, District of Columbia; ED, emergency department; FL, Florida; GA, Georgia; IL, Illinois; KS, Kansas; KY, Kentucky; LA, Louisiana; LOS, length of stay; MA, Massachusetts; MD, Maryland; MI, Michigan; MS, Mississippi; NJ, New Jersey; NM, New Mexico; NY, New York; OH, Ohio; PA, Pennsylvania; SC, South Carolina; TN, Tennessee; TX, Texas; VA, Virginia; WI, Wisconsin.

^a^ Other race include American Indian, Asian, Native Hawaiian or Pacific Islander, White, and other, as reported in the database.

^b^ States with cell counts fewer than 11 were grouped by location.

^c^ Includes direct admissions and admissions from the ED.

## Discussion

This cohort study is the first study, to our knowledge, to use real-world, longitudinal data to redefine high ED use for individuals with SCD. Strengths of this study include the use of 7 years of data from the North Carolina Hospital Discharge Data, representing 9964 individuals with SCD. The data are compelling, and we were able to further refine the definition of high ED utilization, which previously has been described as greater than 3 to 6 ED visits per year. We propose a new definition of ED utilization for SCD. Using the distribution of people based on the number of annual ED visits over a 7-year period, we defined 4 categories of utilization: low, 0 to 1 ED visits in all years; moderate, 2 to 9 visits in any year; high, 10 to 32 visits in any year; and super high, 33 or more ED visits in any year. Had we used the previous definitions of high or super-high ED utilization with the cutoff of 3 or more visits, approximately 66% of persons in the cohort would have been categorized as high users. Our clinical experience that few patients have high ED utilization was confirmed. Only 1.7% of individuals with SCD had super-high ED utilization; some patients contributed more than 200 visits in a year. Super-high utilization was not consistent across the years for individual patients. These data reinforce the experience of ED physicians and may contribute to the perception of addiction among individuals with SCD. ED clinicians are less likely to remember the low number of visits from the 3397 patients with 0 to 1 ED visits per year; rather, they are more likely to remember patients in the high and super-high utilization categories. Patients ages 19 to 39 vs younger than 19 years were more likely to be in the moderate, high, or super-high utilization cohorts, as described in previous reports of ages 19 to 35 years,^3^18 to 30 years,^28^ 20 to 29 years,^20^ older than 15 years,^19^ and older than 21 years.^21^ Increased ED visits during transition from pediatric to adult care represents a high-risk period and identifies a vulnerable age group that would benefit from early intervention. Unlike previously reported data,^29^ we did not find differences in sex between utilization cohorts in our sample. Statistically significant differences in race and ethnicity reported in our data were not meaningful, due to the low numbers in the cohort.

Individuals in the moderate, high, and super-high utilization categories were more likely to die compared with the low utilization category, and individuals in our high and super-high categories who died were younger. While we were unable to measure it, disease severity may have contributed to early death. Previous research has identified an association between high ED utilization among patients with more severe disease and pain,^9,16^ including the need for more transfusions and experiencing worse physical function.^9^ It is important for health care practitioners caring for people with super-high ED utilization to consider the increased rate of complications in these patients, facilitate rapid medical evaluation and treatment, and determine a disposition plan.

Most patients in the cohort had a high SVI, especially the themes of household characteristics and housing type and transportation. Previous studies have reported that most patients with SCD relied on family, friends, or public transportation and had to wait for transportation to the ED. Several stated they could not drive because of pain.^30^ Housing, utility, and food insecurity have been also associated with increased ED reliance in pediatric patients, as has being a single mother.^31,32^ Previous screening of patients with SCD in the ED identified the following needs: chronic pain management, other medical needs, emotional, financial (insurance), and assistance with transportation and prescriptions.^33^ Linking patients to more definitive medical or social behavioral health resources may help better address the needs of patients with SCD, especially those with frequent ED utilization. A very high proportion of patients in the high and super-high utilization categories visited multiple EDs during the study period, 80% and 94%, respectively. Routinely visiting multiple EDs requires further attention. These patients likely have some unmet medical, social, or behavioral health need. In addition to acute and chronic pain, individuals have complex medical histories and should be linked to a comprehensive sickle cell center and a primary care practitioner.

To our knowledge, our data are the first to identify a large number of patients from different states with ED visits in North Carolina. Patients from 25 different states presented to EDs in North Carolina. The most common reason for ED visits is treatment of VOE. Individualized pain plans (IPPs) have been recommended to treat VOE. When individuals with SCD experience a VOE in another facility or state outside their SCD medical home, patients are typically subject to poor treatment of their pain. The data from our project emphasize the importance of developing IPPs made available in the electronic medical record and patient portal for ease of access to both ED clinicians and patients. IPPs or use of a weight-based protocol have been recommended by National Heart, Lung, and Blood Institute and the American Society of Hematology for treatment of VOEs.^34,35^ Use of IPPs has been found to be feasible and acceptable to both patients with SCD and ED clinicians.^36^ Placing IPPs in the electronic health record and patient portal for both ED clinicians and patients can facilitate care treatment of VOE in any ED.^36^

There are many implications associated with expanding the categories of what is considered high ED utilization. Moderate utilization (2-9 ED visits) was common, whereas as high and super-high utilization were less common. These categories can help guide clinician-patient conversations. As ED visits begin to increase, there is an opportunity for hematologists, social workers, and patients to work together to identify the causes of moderate ED use. As the visits approach the high and super high categories, ED clinicians should be brought into the conversation, and specific plans should be available in the electronic health record to guide ED management. Often, these patients will have complex medical, social, and behavioral health needs. Payers, particularly Medicaid, should be involved in discussions to ensure necessary services, such as transportation, social, and behavioral health needs, are covered.

### Limitations

This study has some limitations. Medicaid was not expanded in North Carolina until 2022; therefore, we could not examine changes in utilization prior to and after expansion. We were unable to examine previously identified risk factors for high ED utilization, including clinical characteristics representative of both medical (acute chest syndrome, transfusions) and psychiatric (anxiety, depression) morbidity or chronic opioid therapy use. The dataset was limited to *ICD-9-CM* and *ICD-10-CM* codes, which lack the specificity to determine disease complications or severity. We could not measure access to the health care system other than by distance to comprehensive sickle cell care and insurance coverage. Our analysis of insurance data was analyzed per visit instead of the individual patient, thus limiting our ability to study the association between ED utilization and insurance status. It is likely that individuals with more than 20 ED visits in any year had more severe disease and thus more often qualified for both Medicaid and Medicare coverage (dual eligible). Because the North Carolina Hospital Discharge Data do not include outpatient visits, patients with less severe disease and low acute care utilization may be underrepresented. The analysis was limited to hospital discharge data from 1 state, limiting the generalizability of findings. Participants’ SVI scores were reported at the county level instead of the US Census Bureau tract level, potentially obscuring variations in SVI within individual counties. Furthermore, this was a limited dataset, and individuals were identified based on SCD diagnosis, date of birth, zip code, and sex. Patients who moved during the study period may have been counted twice.

## Conclusions

In this cohort study, we determined categories of ED utilization based on real-world longitudinal data, resulting in 4 categories: low, moderate, high, and super high. These categories are drastically different than previous definitions of 3 to 6 ED visits per year. Individuals in the moderate, high, and super-high categories were older, more likely to visit multiple EDs, had higher SVI, and, among those who died, they died younger. These 4 new categories of ED utilization in SCD could be used to reframe how high ED use is determined.

## Notes

Supplement 1. **eFigure.** SCD Cohort Study Inclusion/Exclusion Flow Diagram

Supplement 2.
Data Sharing Statement
