# Supplementary material for: Redefining High Emergency Department Utilization for Sickle Cell Disease
Source: JAMA Netw Open. 2025 Jun 2;8(6):e2513361. doi: 10.1001/jamanetworkopen.2025.13361 (PMC12131098; doi:10.1001/jamanetworkopen.2025.13361)
Supplement: Supplement 1. — eFigure. SCD Cohort Study Inclusion/Exclusion Flow Diagram [file jamanetwopen-e2513361-s001.pdf]

Supplemental Online Content

Tanabe P, Pan W, Blewer AL, et al. Redefining high emergency department utilization for sickle cell disease. *JAMA Netw Open*. 2025;8(6):e2513361. doi:10.1001/jamanetworkopen.2025.13361

eFigure. SCD Cohort Study Inclusion/Exclusion Flow Diagram

This supplemental material has been provided by the authors to give readers additional information about their work.

© 2025 Tanabe P et al. *JAMA Network Open*.

eFigure. SCD Cohort Study Inclusion/ Exclusion Flow Diagram

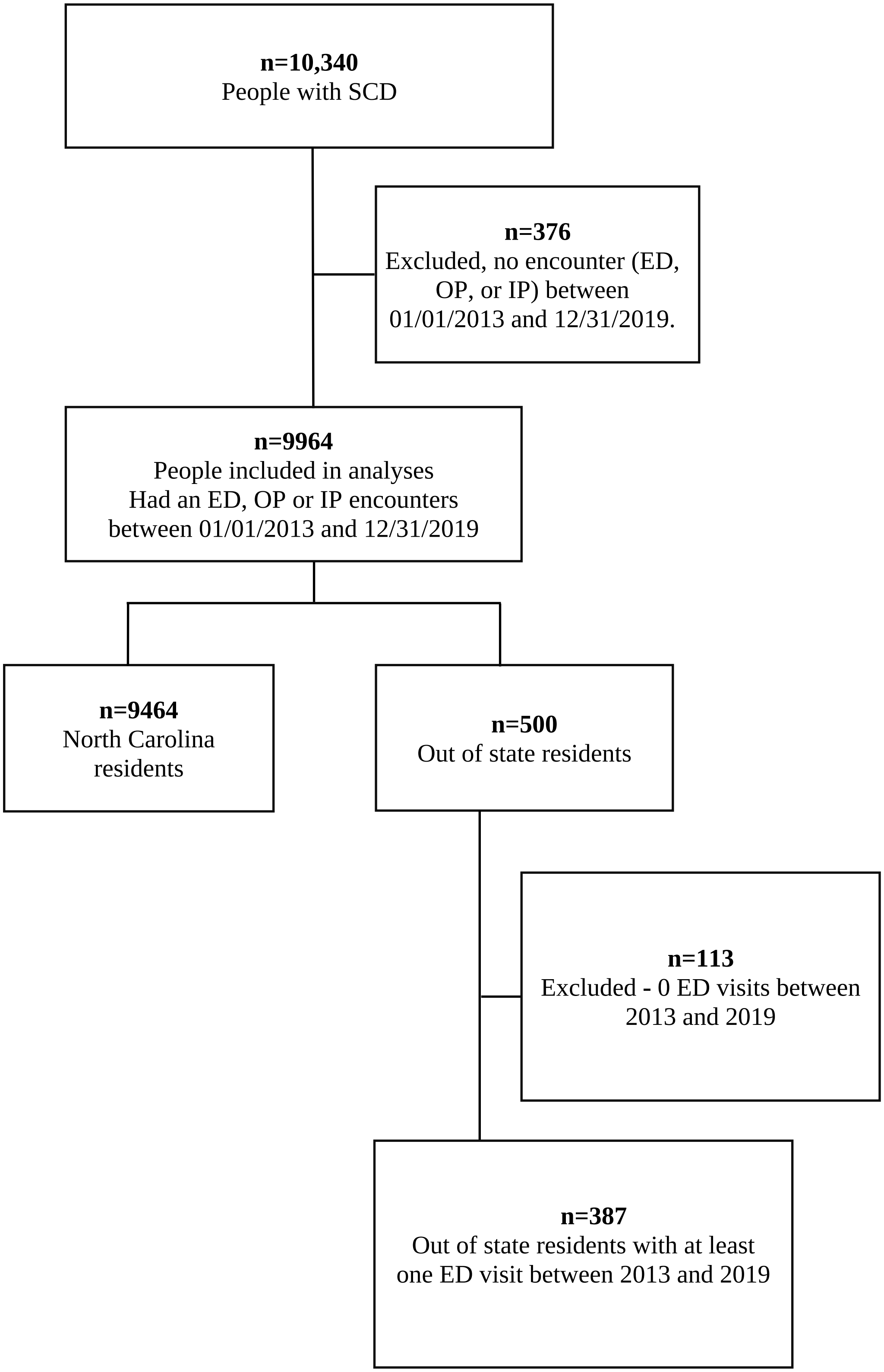

Legend: Abbreviations: SCD=sickle cell disease, ED= emergency department, OP= outpatient surgery, IP=inpatient admission

© 2025 Tanabe P et al. *JAMA Network Open*.
