# Supplementary material for: Redefining High Emergency Department Utilization for Sickle Cell Disease
Source: JAMA Netw Open. 2025 Jun 2;8(6):e2513361. doi: 10.1001/jamanetworkopen.2025.13361 (PMC12131098; doi:10.1001/jamanetworkopen.2025.13361)
Supplement: Supplement 2. — Data Sharing Statement [file jamanetwopen-e2513361-s002.pdf]

Data Sharing Statement

Data
Data available: No

Additional Information
Explanation for why data not available: The data supporting the findings of this study are available within the article and/or its supplementary materials; since participants did not give written consent and in agreement with the North Carolina Sickle Cell Data Collection program data use arrangements line level data cannot be shared publicly.
